# Patient Reported Outcome Measure in Atopic Dermatitis Patients Treated with Dupilumab: 52-Weeks Results

**DOI:** 10.3390/life11070617

**Published:** 2021-06-25

**Authors:** Servando E. Marron, Lucia Tomas-Aragones, Carlos A. Moncin-Torres, Manuel Gomez-Barrera, Francisco Javier Garcia-Latasa de Aranibar

**Affiliations:** 1 Dermatology Department, University Hospital Miguel Servet, Aragon Psychodermatogy Research Group (GAI+PD), Paseo de Isabel la Catolica 1-3, 50009 Zaragoza, Spain; semarron@salud.aragon.es; 2 Psychology Department, University of Zaragoza, Aragon Psychodermatology Research Group (GAI+PD), c/Pedro Cerbuna 12, 50009 Zaragoza, Spain; 3 Pharmacy Department, Royo Villanova Hospital, Avda. San Gregorio 30, 50015 Zaragoza, Spain; camoncin@salud.aragon.es; 4 Health Sciences Faculty, San Jorge University, Zaragoza, Autovia Mudejar, Km 299, Villanueva de Gallego, 50830 Zaragoza, Spain; mgomezbarrera@gmail.com; 5 Pharmacoeconomics & Outcomes Research Iberia (PORIB), Paseo de Joaquín Rodrigo 4-I, Pozuelo de Alarcón, 28224 Madrid, Spain; 6 Dermatology Department, Royo Villanova Hospital, Aragon Psychodermatology Research Group (GAI+PD), Avda. San Gregorio 30, 50015 Zaragoza, Spain; fjgarcial@salud.aragon.es

**Keywords:** atopic dermatitis, patient reported outcome measures, dupilumab, quality of life, satisfaction, efficacy, safety, adherence

## Abstract

Dupilumab is used to treat atopic dermatitis (AD) patients who have proven to be refractory to previous treatments. The aim of this study was to assess evolution and patient reported outcome measures in adult patients with moderate-to-severe AD treated with dupilumab in routine clinical practice. The outcomes were evaluated and registered at baseline and weeks 16, 40 and 52. The variables evaluated were: disease severity, pruritus, stressful life events, difficulty to sleep, anxiety and depression, quality of life, satisfaction, adherence to the treatment, efficacy and safety. Eleven patients were recruited between 14 Nov 2017 and 16 Jan 2018. Demographic variables: 90% Caucasian, 82% women. Clinical variables: Mean duration of AD = 17.7 (±12.8), 91% had severe disease severity. At baseline, SCORAD median (range) score = 69.2 (34.8–89.2); itch was reported by 100% of patients; itch visual analogue scale median (range) was 9 (6–10); HADS median (range) total score = 13 (5–21); DLQI mean score = 16 (2–27); EQ-5D-3L median (range) = 57 (30–99). At week-52 there was a significant reduction of SCORAD scores median (range) = 4.3 (0–17.1), HADS total score median (range) = 2 (0–10) and improved quality of life EQ-5D-3L median (range) = 89 (92–60). This study confirms that dupilumab, used for 52-weeks under routine clinical practice, maintains the improved atopic dermatitis signs and symptoms obtained at week 16, with a good safety profile.

## 1. Introduction

Atopic dermatitis (AD), a chronic inflammatory skin disease that evolves as flares, is characterized by itching and eczema. AD affects 2–10% of adults [1] and, in severe cases, is associated with significant psychosocial distress [2]. Moderate-to-severe AD requires long-term immunosuppressive therapy of which the efficacy profile and long-term adverse effects are not clear [3,4,5,6]. Treatment guidelines recommend short-term high-strength topical corticosteroids with or without calcineurin inhibitors to control outbreaks, in addition to topical emollients [7,8]. Systemic treatment with corticosteroids or immunosuppressants (cyclosporine, methotrexate, azathioprine, etc.), and phototherapy are indicated only in cases not controlled by topical treatment [9,10]. Long-term systemic treatments are not indicated due to their bad safety and efficacy profile [11,12].

Dupilumab is a human monoclonal antibody that specifically blocks the alpha receptor of interleukin-4 (IL-4), inhibiting interleukin-4 and interleukin-13 signaling of these inflammatory cytokines involved in various allergic diseases, such as asthma, atopic dermatitis and rhinitis [13]. Dupilumab has shown a good efficacy and safety profile in monotherapy or associated with topical corticosteroids [14,15,16]. It has been approved by the European Medicines Agency [17] for the treatment of moderate-to-severe AD in adult patients in whom systemic treatment is indicated.

Patient-reported outcome measure (PROMs) provide an important adjunct to clinician-assessed measures in atopic dermatitis [18].

The aim of this study is to assess evolution, security and PROMs in adult patients with moderate-to-severe atopic dermatitis treated with dupilumab in routine clinical practice. Efficacy, safety, psychosocial impact, quality of life, adherence to treatment, security and satisfaction were the variables measured in adult patients with AD treated with subcutaneous dupilumab during the 52 weeks. The analysis of preliminary data in week 16 were previously published [19]. We present definitive data up to week 52.

## 2. Materials and Methods

### 2.1. Study Group

This study included AD patients who met the criteria for dupilumab treatment in the Dermatology department of the Royo Villanova Hospital, Zaragoza, Spain, between 14th November 2017 and 16th January 2018. Treatment with dupilumab was possible through adherence to a program of extended medication use authorized by the Spanish Agency for Medicine and Health Products (AEMPS). All patients gave signed informed consent to be included in the program and authorized the use of the clinical data obtained. The hospital pharmacy service sent a request and individualized report on the patient to be included in the treatment program to AEMPS. Once authorization was obtained, the pharmaceutical company sent the medication for each patient to the pharmacy service for dispensation. Patients had to apply emollient creams twice a day after the baseline visit (request for inclusion). Once treatment had been authorized and the medication was available in the hospital pharmacy, treatment with dupilumab 300 mg subcutaneously every 2 weeks, with an initial loading dose of 600 mg on the first day, was administered. To prevent conjunctivitis, patients were recommended to use preventive artificial tears. The following face-to-face visits were included in the protocol: inclusion, treatment initiation (baseline visit), weeks 4, 8, 12, 16 and then every 12 weeks (weeks 28, 40 and 52). A Case Report Form (CRF) was completed for each treatment visit to guarantee data quality. The sociodemographic variables collected were: age, gender, education, family status and occupation. Other variables: stressors during the previous 6 months, height, weight and body mass index (BMI). Routine clinical demographic data included: age at onset of AD, years of evolution, patient-perceived severity in the last year and currently, and previous topical and systemic treatments administered. The study was carried out according to the provisions of the Helsinki Declaration and current Spanish legislation. All patients were evaluated and authorized individually by AEMPS; it was necessary to renew this authorization every 12 weeks for treatment to continue. The results analyzed were obtained during the baseline visit and at 16, 40 and 52 weeks of treatment.

### 2.2. Outcome Variables

In order to evaluate the influence of AD on patients’ outcome variables, they were asked to complete: (a) AD severity using the Scoring Atopic Dermatitis (SCORAD) index [20]; (b) variables related to pruritus (stinging, burning or pain) and its negative impact on their relationships, sleep disturbance and mood, and pruritus level according to a Visual Analogue Scale (VAS); (c) anxiety and depressive symptoms using the Hospital Anxiety and Depression Scale (HADS) [21]; (d) quality of life using the EuroQol 5D-3L (EQ-5D-3L) [22] and the Dermatology Life Quality Index (DLQI) [23]; (e) patient satisfaction using an “ad hoc” (VAS) [24] and the Consumer Reports Effectiveness Scale (CRES-4) [25], which measures satisfaction with treatment; (f) treatment adherence, checked by counting boxes and syringes consumed at each visit; (g) safety; and (h) adverse effects. The results are obtained from the analysis of the comparison at the baseline visit, and at 16, 40 and 52 weeks.

### 2.3. Statistical Analysis

Qualitative variables are shown as frequencies and percentages and quantitative variables as mean, median, standard deviation (SD), maximum and minimum. VAS variables were considered discrete quantitative variables and the median and range were calculated. Comparisons were made between the 3 moments of ANOVA for repeated measures, and Student’s *t* test for related data when the variable was distributed normally or with the Friedman or Wilcoxon signed-rank tests when it was not. Normality was determined using the Kolmogorov–Smirnov test. A value of *p* = 0.05 was used as a threshold to accept or reject the null hypotheses; however, this p-value was 0.016 (0.05/3) to make the bivariate comparison between the three moments after rejecting the hypotheses of equality between the three moments. The data were analyzed using SPSS 25.0.

## 3. Results

The study group comprised of 11 adult patients, of which nine (81.8%) were females; six (54.5%) had secondary studies and five (45.5%) had university studies; eight lived with family (72.7%) and three (27.3%) alone; three were students (27.3%), five (45.5%) were active workers, one (9.1%) was unemployed and one (9.1%) was on sick leave; five patients (45.5%) reported having had stressful life events in the last six months. The mean age (± standard deviation, SD) was 33.2 ± 15.6 years with a median 24 and range (71.0–21.0), the mean weight was 72.0 ± 14.4 kg with median 71.0 and range (99.0–53.0); median height was 165.9 ± 5.6 cm with median 167.0 and range (176.0–155.0); the mean body mass index (BMI) was 26.1 ± 4.5 with a median 25.5 and range (34.6–21.1). The mean number of years of illness was 16.6 ± 22.5 with a median 4.0 and range (1.0–66.0); the mean number of years of evolution was 17.7 ± 12.8 with a median 19.0 and range (1.0–44.0); SCORAD mean was 61.7 ± 15.5 with median 65.6 and range (66.0–86.1). A total of 10 (90.9%) patients manifested “severe” type and one (9.1%) “moderate” type. Regarding concomitant treatments, all patients received emollients twice a day and artificial tears.

### 3.1. Evolution of Visits: Baseline, 16, 40 and 52 Weeks

The evolution of the variables related to the symptoms are presented below in Table 1. All the patients had severe lesions at baseline and practically no lesions in week 52. Variables related to the evolution of the EQ-5D-3L subscales perceived by patients are presented in Table 2, showing that all the patients were without mobility problems and personal care problems in week 52. Variables related to safety are presented in Table 3 with no incidence of serious adverse events, and variables related to satisfaction between current treatment with dupilumab and previous treatments are presented in Table 4, showing greater satisfaction with dupilumab than with previous treatments.

### 3.2. Comparative of Symptoms Analysis at Baseline, 16, 40 and 52 Weeks

**Figure 1:**
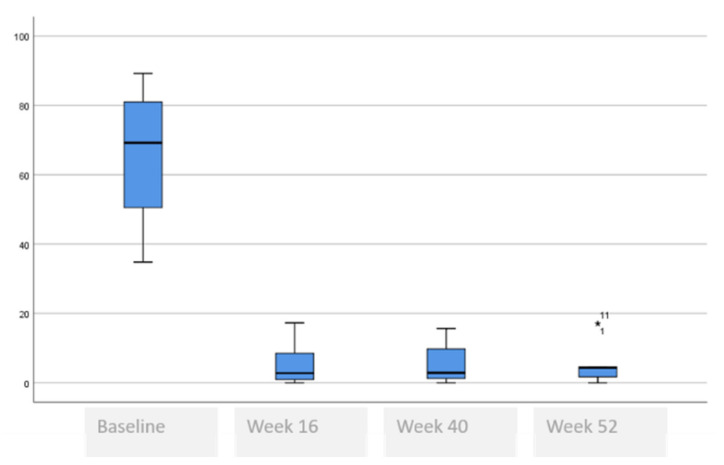
SCORAD evolution.

**Figure 2:**
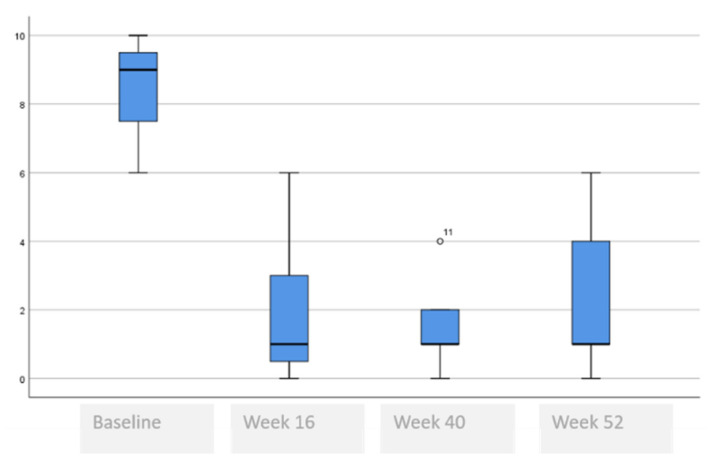
Evolution of itch assessment.

**Figure 3:**
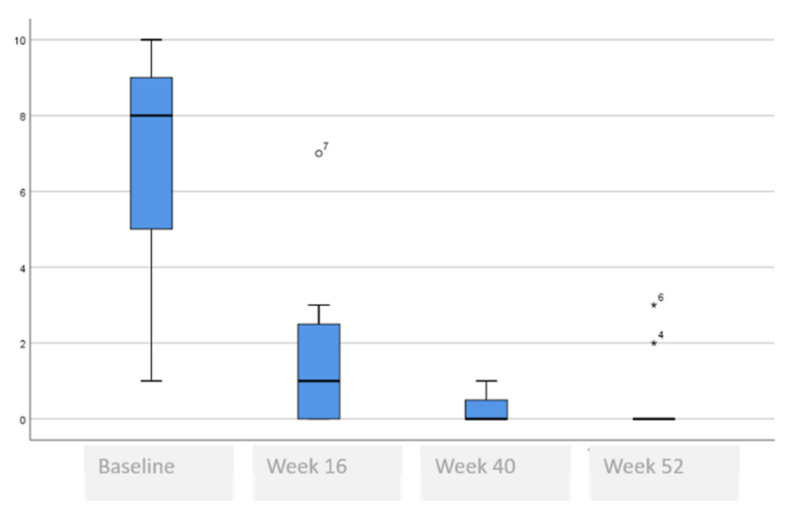
Evolution of difficulty sleeping.

The following tables and figures present the comparison of the baseline visit with week 16, week 40 and week 52. Table 5 and Figure 1, Figure 2 and Figure 3 show the evolution of the disease severity and symptoms, showing a decrease of disease severity (SCORAD Index) from (64.5 ± 19.6 to 5.5 ± 5.9, *p* < 0.001) at week 16 that was maintained or even improved in weeks 40; (5.8 ± 5.7) and week 52; (5.3 ± 6.0). Regarding pruritus, a decrease was observed between the baseline visit and week 16 from 8 to 1, (*p* < 000.1), that was maintained in week 40 and week 52, although a slightly higher value was observed in the itch in week 52 which was not statistically significant, (*p* = 0.259). Regarding difficulty to sleep, a decrease was also observed at week 16 from 8 to 1 (<0.001), that showed a tendency to improve until it stopped being a problem at week 44 and week 52.

### 3.3. Comparative Self-Perceived Psychological and Quality of Life Issues Analysis at Baseline, 16, 40 and 52 Weeks

**Figure 4:**
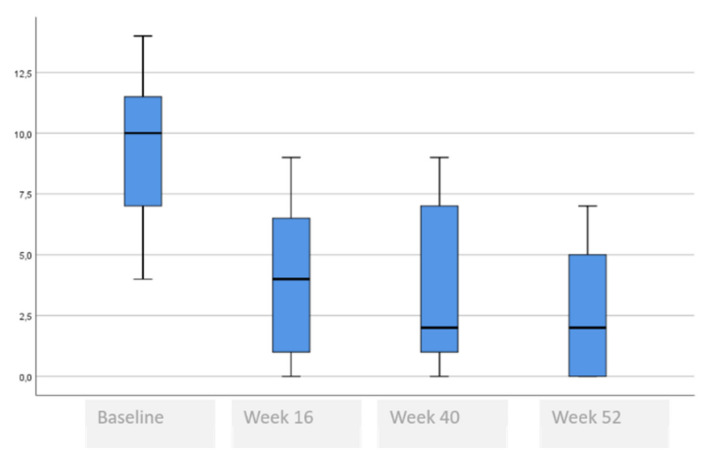
Evolution of the HADS anxiety score.

**Figure 5:**
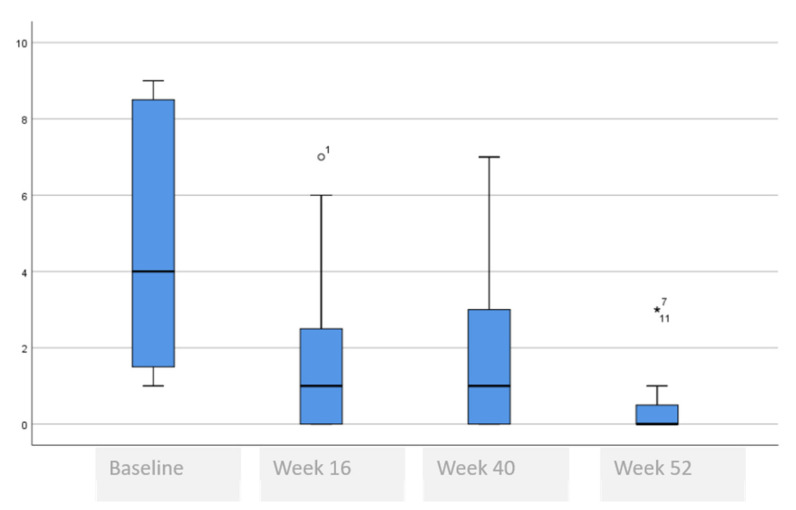
Evolution of the HADS depression score.

**Figure 6:**
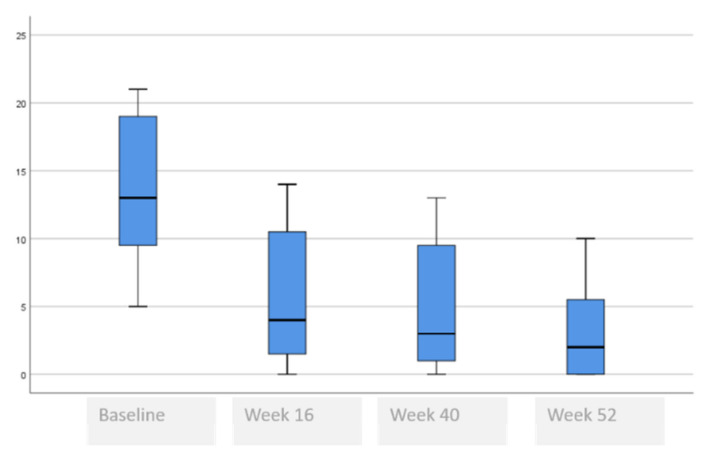
Evolution of the total HADS score.

**Figure 7:**
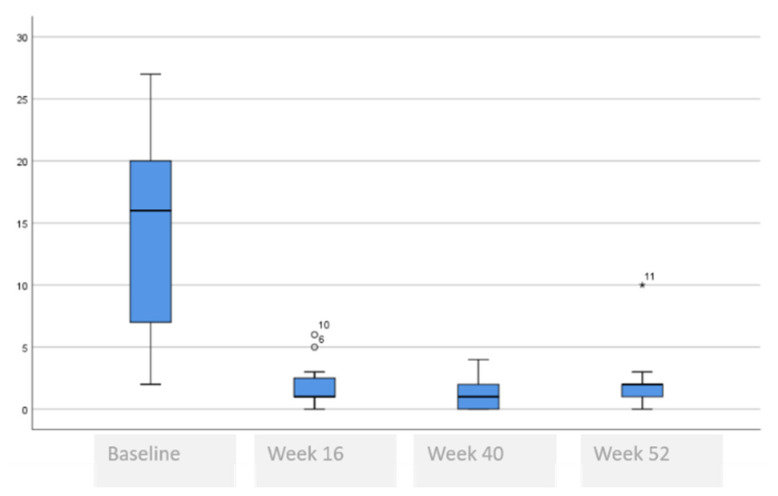
DLQI score evolution.

**Figure 8:**
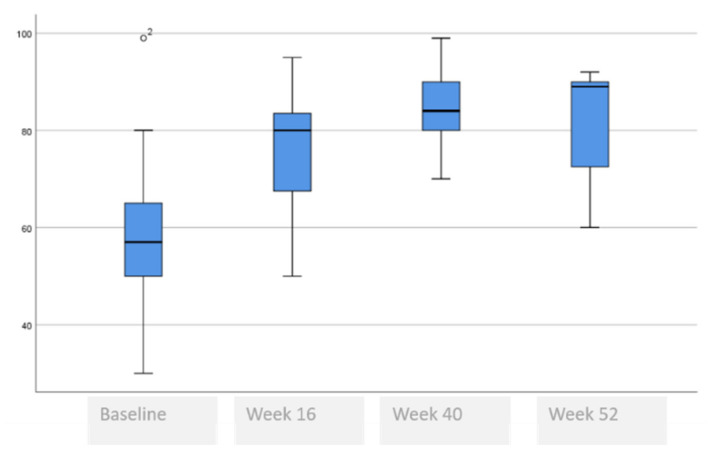
Evolution of the EQ-5D-3L.

Table 6 and Figure 4, Figure 5, Figure 6, Figure 7 and Figure 8 show the evolution of quality of life, which improved at week 16 and remained unchanged until week 52.

HADS score fell by (9.2 ± 3.0 to 3.9 ± 3.4, *p* = 0.007) at week 16 and continued to decline at week 40 and week 52 (<0.001), and for depression fell by (4.7 ± 3.4 to 1.9 ± 2.5, *p* = 0.008) at week 16 and continued to decline at week 40 and week 52 (<0.001). In the DLQI questionnaire, scores of (13.9 ± 8.3 to 2.0 ± 1.8, *p* = 0.001) at week 16 continued to decline at week 40 and week 52 (<0.001). In the EQ-5D-3L, VAS increased from 57 to 80 (*p* = 0.035) at week 16, and continued to increase during week 40 and week 52 (<0.001).

### 3.4. Comparative Satisfaction Analysis at Baseline, 16, 40 and 52 Weeks

The values referred to the comparison of the CRES-4 score are presented in Table 7 There are no statistically significant differences between week 16, week 40 and week 52.

The results of the comparison of satisfaction with the current treatment compared to the previous one at week 16, and at week 40 with week 16 and 52 are presented in Table 8. Satisfaction increased from week 16, although there was a reduction in global satisfaction between weeks 40 and 52. This reduction is more statistical than clinical; the descriptive values are the same (median equal to 10), producing some difference in the range, which drops the minimum value from 7 to 4.

## 4. Discussion

The results of this study show that dupilumab 300 mg q2w in monotherapy promotes a rapid and sustained statistically significant improvement in the SCORAD score of adults with moderate to severe atopic dermatitis at week 16, which is maintained until week 52. The improvement in the SCORAD score is comparable to previous studies performed: the 2017 SOLO 1 and SOLO 2 studies [16] at week 16; the 2018 phase III LIBERTY AD CAFÉ trial [26] where dupilumab every two weeks reduced the SCORAD index by 62.4%, compared with 91% in our study; the 52- week LIBERTY AD CHRONOS study had a cut-off point at 16 weeks, with which our results may be compared [27]. At week 16, there was a significant reduction in the SCORAD index of 62.1%. A polled analysis of a phase 2a and a phase 2b study and a sub-analysis of the 2b study [28] also show a significant improvement of SCORAD at week 12. In a real-life multicenter study [29], a 3 month follow-up showed a significative reduction in the SCORAD score, and in a post hoc analysis Barbarot et al. [30] included 2444 patients in four placebo-controlled, double-blind, randomized, phase 3 trials. SOLO 1 and SOLO 2 evaluated 16 weeks of dupilumab monotherapy against a placebo. CAFÉ and CHRONOS evaluated dupilumab with concomitant topical corticosteroids (TCS) against TCS alone for 16 and 52 weeks, respectively, and published SCORAD scores show significative reduction at both week 16 and week 56, respectively, similar to our results. Tofte et al. [28] report a significant and rapid improvement of pruritus reduction in the first week, and it continued to decrease its intensity until week 12, data similar to that observed in our sample in week 4, which were maintained until week 52. Similarly, de Bruin-Weller et al. [26] document a reduction in pruritus, measured as the percentage of patients achieving reductions of >4 points on the NRS scale, compared with a reduction of 7 points in the VAS for itching found in our study (*p* = 0.003) at week 16. In addition, Blauvelt et al. [27] evaluated itching as a secondary outcome, obtaining significant reductions, in a similar way to those obtained by us. Additionaly, Simpson et al. [16] show an improvement in pruritus symptoms in the SOLO-1 and SOLO-2 studies. These results cannot be compared with ours, however they do support our findings. In addition to its positive effect on itch, improvement in sleep was observed in patients treated with dupilumab, with a reduction in the VAS difficulty-in-sleeping scale from 8 to 1 (*p* = 0.006), and disappearing at week 40 and week 52 (*p* < 0.001), similar to the results reported by Simpson et al. in 2016 [15] who measured improvements by a VAS reduction of 3.7 and the POEM questionnaire. Tofte et al. [28] observed, in a pooled analysis of two phase 2 clinical trials, a rapid and significant reduction (week 2) in sleep disturbance that was maintained until week 12 (*p* < 0.05 vs. placebo). The 52-week LIBERTY AD CHRONOS study had a cut-off point at 16 weeks, with which our results may be compared [27]. At week 16 there was a significant reduction in the DLQI of 9.7 points, and in the HADS of 4.9 which, as previously mentioned, is consistent with previously published results. In the 2018 phase III LIBERTY AD CAFÉ trial [26], dupilumab every two weeks reported a mean reduction of 6.1 in the HADS index, in line with the 5.8 reduction in our study at week 16, and a reduction of 9.5 in the DLQI score [28], similar to the 11.9 reduction in our study. Tofte et al. [28] also reported mild or moderate adverse effects, similar to those obtained by us, except for conjunctivitis. In the two clinical trials, they report 9.3% and 1.7%, respectively, very divergent data between one and the other, which differ from our data in which we did not observe conjunctivitis, probably due to our precaution in the use of artificial tears at the beginning of the treatment and maintaining their use throughout.

We also analyzed satisfaction with treatment, measured using the validated CRES-4 scale [25]. The results showed good satisfaction with treatment, both clinically and in communication with the physician, and the emotional perception of the treatment. The perception of satisfaction was also assessed with a series of “ad hoc” questions made using VAS, among which the score of 4 out of 10 in relation to previous treatments and 9 out of 10 with dupilumab stand out, both in week 16, and at week 40 and week 52 (<0.001). This is an important contribution of the present study, as this factor has not been previously reported.

Our study had some relevant limitations. It was a single-center study carried out in a small number of patients, which makes the external validation complicated. The strengths of the study are the extensive data collection and controls to guarantee quality, and the assessment of patients’ satisfaction with the treatment.

## 5. Conclusions

In our study we have observed in week 52 that dupilumab is effective in the treatment of adult patients with moderate to severe AD, rapidly reducing the signs and symptoms of AD and improving psychological impact and quality of life. The safety profile was excellent and we did not observe any cases of conjunctivitis, probably due to the preventive use of artificial tears during treatment, whose use we recommend. Adherence to treatment and perception of satisfaction showed that patients valued dupilumab treatment significantly more than previous treatments.

## Figures and Tables

**Table 1:** Symptom descriptions at baseline visits, 16, 40 and 52 weeks.

Variable	Baseline	Week 16	Week 40	Week 52
Severity, n (%)				
Without lesions	0 (0.0)	3 (27.3)	2 (18.2)	4 (36.4)
Almost no lesions	0 (0.0	2 (18.2)	8 (72.7)	6 (54.5)
Mild	0 (0.0)	4 (36.4)	1 (9.1)	1 (9.1)
Moderate	0 (0.0)	2 (18.2)	0 (0.0)	0 (0.0)
Severe	11(100)	0 (0.0)	0 (0.0)	0 (0.0)
Weight, mean (SD)	74.7 (14.2)	74.0 (15.3)	75.4 (13.6)	75.0 (15.8)
BMI, mean (SD)	27.0 (4.4)	26.8 (4.9)	27.2 (4.2)	27.1 (5.0)
SCORAD, mean (SD)	64.5 (19.6)	5.5 (5.9)	5.8 (5.7)	5.3 (6.0)
Itching; Yes, n (%)	11 (100)	8 (72.7)	7 (63.6)	9 (81.8)
VAS pruritus, median (range)	8 (6–10)	1 (0–6)	1 (0–4)	1 (0–6)
Pruritus, characteristics; Yes, n (%)				
Itching only	4 (36.4)	9 (81.8)	9 (81.8)	9 (81.8)
Burning	8 (72.7)	0 (0.0)	0 (0.0)	0 (0.0)
Stinging	8 (72.7)	0 (0.0)	0 (0.0)	0 (0.0)
Pain	7 (63.6)	0 (0.0)	0 (0.0)	0 (0.0)
Pruritus, frequency; Yes, n (%)				
Never	0 (0.0)	2 (18.2)	1 (9.1)	2 (18.2)
Rarely	0 (0.0)	3 (27.3)	6 (54.5)	6 (54.5)
Sometimes	1 (9.1)	4 (36.4)	4 (36.4)	2 (18.2)
Often	5 (45.5)	2 (18.2)	0 (0.0)	1 (9.1)
Always	5 (45.5)	0 (0.0)	0 (0.0)	0 (0.0)
Unbearable itching, n (%)				
Never	0 (0.0)	6 (54.5)	7 (63.6)	7 (63.6)
Rarely	0 (0.0)	3 (27.3)	1 (9.1)	3 (27.3)
Sometimes	3 (27.3)	2 (18.2)	2 (18.2)	1 (9.1)
Often	3 (27.3)	0 (0.0)	1 (9.1)	0 (0.0)
Always	5 (45.5)	0 (0.0)	0 (0.0)	0 (0.0)
Impact of pruritus on others, n (%)				
Never	2 (18.2)	9 (81.8)	7 (63.6)	8 (72.7)
Rarely	0 (0.0)	2 (18.2)	2 (18.2)	2 (18.2)
Sometimes	1 (9.1)	0 (0.0)	2 (18.2)	1 (9.1)
Often	5 (45.5)	0 (0.0)	0 (0.0)	0 (0.0)
Always	3 (27.3)	0 (0.0)	0 (0.0)	0 (0.0)
Impact of pruritus on sleep, n (%)				
Never	0 (0.0)	7 (63.6)	8 (72.7)	7 (63.6)
Rarely	1 (9.1)	4 (36.4)	1 (9.1)	3 (27.3)
Sometimes	1 (9.1)	0 (0.0)	2 (18.2)	1 (9.1)
Often	5 (45.5)	0 (0.0)	0 (0.0)	0 (0.0)
Always	4 (36.4)	0 (0.0)	0 (0.0)	0 (0.0)
Impact of pruritus on mood, n (%)				
Never	0 (0.0)	8 (72.7)	7 (63.6)	9 (81.8)
Rarely	2 (18.2)	3 (27.3)	2 (18.2)	1 (9.1)
Sometimes	1 (9.1)	0 (0.0)	2 (18.2)	1 (9.1)
Often	6 (54.5)	0 (0.0)	0 (0.0)	0 (0.0)
Always	2 (18.2)	0 (0.0)	0 (0.0)	0 (0.0)
Difficulty sleeping; Yes, n (%)	11 (100)	6 (54.5)	1 (9.1)	2 (18.2)
VAS difficulty sleeping, median (range)	8 (1–10)	1 (0–7)	0 (0–1)	0 (0–3)

SD: Standard deviation; VAS: Visual analogue scale; BMI: Body mass index; SCORAD: Scoring atopic dermatitis.

**Table 2:** Descriptive variables of EQ-5D-3L subscales at baseline visits, 16, 40 and 52 weeks.

Variable	Baseline	Week 16	Week 40	Week 52
EQ-5D-3L mobility problems, n (%)				
None	11 (100)	11 (100)	11 (100)	11 (100)
Some	0 (0.0)	0 (0.0)	0 (0.0)	0 (0.0)
Many	0 (0.0)	0 (0.0)	0 (0.0)	0 (0.0)
EQ-5D-3L personal care problems, n (%)				
None	9 (81.8)	11 (100)	11 (100)	11 (100)
Some	2 (18.2)	0 (0.0)	0 (0.0)	0 (0.0)
Many	0 (0.0)	0 (0.0)	0 (0.0)	0 (0.0)
EQ-5D-3L problems in daily activities, n (%)				
None	6 (54.5)	11 (100)	100 (0.0)	11 (100)
Some	5 (45.4)	0 (0.0)	0 (0.0)	0 (0.0)
Many	0 (0.0)	0 (0.0)	0 (0.0)	0 (0.0)
EQ-5D-3L discomfort, pain problems, n (%)				
None	3 (27.3)	10 (90.1)	10 (90.9)	11 (100)
Some	8 (72.7)	1 (9.1)	1 (9.1)	0 (0.0)
Many	0 (0.0)	0 (0.0)	0 (0.0)	0 (0.0)
EQ-5D-3L anxiety/depression problems, n (%)				
None	3 (27.3)	9 (81.8)	9 (81.8)	11 (100)
Some	6 (54.5)	1 (9.1)	2 (18.2)	0 (0.0)
Many	2 (18.2)	1 (9,1)	0 (0.0)	0 (0.0)
EQ-5D-3L VAS, mean (range)	57 (99–30)	80 (95–50)	84 (99–70)	89 (92–60)

DLQI: Dermatology Life Quality Index; SD: Standard deviation; EQ-5D-3L: EuroQol 5 Dimensions; VAS: Visual analogue scale; HADS: Hospital Anxiety and Depression Scale.

**Table 3:** Descriptive of security variables at baseline visits, 16, 40 and 52 weeks.

Variable	Baseline	Week 16	Week 40	Week 52
SBP, mmHg, mean (SD)	127.3 (16.4)	121.8 (14.2)	121.2 (9.7)	128.0 (11.1)
DBP, mmHg, mean (SD)	82.1 (12.4)	78.7 (12.6)	80.0 (10.6)	83.1 (8.4)
Pulse, BPM, mean (SD)	79.0 (17.9)	72.7 (12.6)	76.2 (10.6)	83.7 (7.7)
Temperature, mean °C (SD)	36.1 (10.3)	36.0 (0.3)	36.2 (0.3)	36.1 (0.3)
Local reaction; Yes, n (%)	2 (18.2)	0 (0.0)	0 (0.0)	0 (0.0)
Severity, n (%)				
Mild	2 (18.2)	0 (0.0)	0 (0.0)	0 (0.0)
Moderate	0 (0.0)	0 (0.0)	0 (0.0)	0 (0.0)
Severe	0 (0.0)	0 (0.0)	0 (0.0)	0 (0.0)
General reaction; Yes, n (%)	0 (0.0)	0 (0.0)	0 (0.0)	0 (0.0)
Analytical alteration; Yes, n (%)	0 (0.0)	1 (9.1)	0 (0.0)	0 (0.0)

SD: Standard deviation; DBP: Diastolic blood pressure; SBP: Systolic blood pressure; BPM: Beats per minute.

**Table 4:** Median value of VAS of satisfaction with current and previous treatment.

Variable	Week-16	Dupilumab Week-40	Week-52	Previous
Satisfaction with training received to administer treatment, median (range)	9 (6–10)	9 (6–10)	9 (6–10)	6 (0–10)
Satisfaction with information from dermatologist, median (range)	9 (6–10)	9 (6–10)	9 (6–10)	6 (0–9)
Satisfaction with disease control, median (range)	9 (6–10)	9 (6–10)	9 (6–10)	3 (0–8)
Satisfaction with frequency of administration, median (range)	9 (6–10)	9 (6–10)	9 (6–10)	2 (0–9)
Effectiveness of treatment to prolong time between flares, median (range)	9 (6–10)	9 (6–10)	9 (6–10)	2 (0–9)
Effectiveness of treatment in control of flares, median (range)	9 (6–10)	9 (6–10)	9 (6–10)	2 (0–8)
Overall satisfaction, median (range)	9 (8–10)	9 (8–10)	9 (8–10)	4 (0–10)

VAS: Visual Analog Scale.

**Table 5:** Comparative analysis of disease severity and symptoms at baseline visits, 16, 40 and 52 weeks.

Variable	Baseline	Week 16	Week 40	Week 52	*p* Value
Weight, mean (SD)	74.7 (14.2)	74.0 (15.3)	75.4 (13.6)	75.0 (15.8)	0.993 ^£^
BMI, mean (SD)	27.0 (4.4)	26.8 (4.9)	27.2 (4.2)	27.1 (5.0)	0.994 ^£^
SCORAD, mean (SD)	64.5 (19.6)	5.5 (5.9)	5.8 (5.7)	5.3 (6.0)	<0.001 ^£^ * <0.001 ^Ω^ (baseline vs. rest) *
VAS pruritus, median (range)	8 (6–10)	1 (0–6)	1 (0–4)	1 (0–6)	<0.001 ^¥^ * 0.003 ^§^ (baseline vs. rest) * 0.259 ^§^ (week 40 vs. week 52)
VAS difficulty sleeping, median (range)	8 (1–10)	1 (0–7)	0 (0–1)	0 (0–3)	<0.001 ^¥^ * 0.006 ^§^ (baseline vs. rest) * 0.036 ^§^ (week 16 vs. rest)

^£^: Repeated measures ANOVA; ^¥^: Friedman test; ^Ω^: Baseline Student’s *t* test versus the other visits; ^§^ Wilcoxon test. SD: Standard deviation; VAS: Visual analogue scale; MBI: Body Mass Index. * significant differences.

**Table 6:** Comparative analysis of quality-of-life results as perceived by patients at baseline visits 16, 40 and 52 weeks.

Variable	Baseline	Week 16	Week 40	Week 52	*p* Value
HADS anxiety, mean (SD)	9.2 (3.0)	3.9 (3.4)	3.4 (3.5)	2.6 (2.8)	<0.001 ^£^ * <0.007 ^Ω^ (baseline vs. Week 16) * <0.224 ^Ω^ (baseline vs. Week 52)
HADS depression, mean (SD)	4.7 (3.4)	1.9 (2.5)	2.0 (2.3)	0.6 (1.0)	0.001 ^£^ * <0.008 ^Ω.^ (baseline vs. Week 16) * <0.127 ^Ω.^ (baseline vs. Week 52)
HADS Total, mean (SD)	13.9 (5.5)	5.8 (5.0)	5.4 (5.0)	3.2 (3.9)	0.001 ^£^ * <0.004 ^Ω^ (baseline vs. week 16) * <0.138 ^Ω^ (baseline vs. week 52)
DLQI, mean (SD)	13.9 (8.3)	2.0 (1.8)	1.3 (1.4)	2.1 (2.7)	<0.001 ^£^ * 0.001 ^Ω^ (baseline vs. week 52) * 0.931 ^Ω^ (week 16 vs. week 52)
VAS EQ-5D-3L, median (range)	57 (30–99)	80 (50–95)	84 (70–99)	89 (60–92)	<0.001 ^£^ * 0.035 ^Ω^ (baseline vs. week 16) 0.225 ^Ω^ (week 16 vs. week 52)

^£^: Repeated measures ANOVA; ^Ω^: Student’s *t* test; DLQI: Dermatology Life Quality Index; SD: Standard deviation; EQ-5D-3L: EuroQol 5D-3L; VAS: Visual analog scale; HADS: Hospital Anxiety and Depression Scale. * significant differences.

**Table 7:** Comparative of satisfaction between dupilumab and previous treatment, measured with VAS.

Variable	Previous	Current at Week 16	*p* Value (Previous vs. Current at Week 16)	Current at Week 40	*p* Value (Current vs. Previous at Week 16 and 40)	Current at Week 52	*p* Value (Previous vs. Current at Week 40 and 52)
Satisfaction with training received to administer treatment, median (range)	6 (0–10)	9 (6–10)	0.019 ^§^	9 (8–10)	0.296 ^§^	9 (8–10)	0.811 ^§^
Satisfaction with information from dermatologist, median (range)	6 (0–9)	9 (6–10)	0.01 ^§^	9 (8–10)	0.397 ^§^	9 (8–10)	0.900 ^§^
Satisfaction with disease control, median (range)	3 (0–8)	9 (6–10)	<0.001 ^§^	9 (8–10)	0.140 ^§^	9 (7–10)	0.796 ^§^
Satisfaction with frequency of administration, median (range)	2 (0–9)	9 (6–10)	0.001 ^§^	9 (7–10)	0.395 ^§^	9 (7–10)	0.147 ^§^
Effectiveness of treatment to prolong time between flares, median (range)	2 (0–9)	9 (6–10)	<0.001 ^§^	9 (6–10)	0.733 ^§^	9 (5–10)	0.362 ^§^
Effectiveness of treatment in control of flares, median (range)	2 (0–8)	9 (6–10)	<0.001 ^§^	9 (4–10)	0.887 ^§^	9 (7–10)	0.104 ^§^
Overall satisfaction, median (range)	4 (0–10)	9 (8–10)	0.001 ^§^	9 (7–10)	0.640 ^§^	9 (4–10)	<0.001 ^§^ *

^§^: Student´s *t* test of related data. * Significant differences.

**Table 8:** Evolution of the CRES-4 score.

Variable	Week 16	Week 40	Week 52	*p* Value
Satisfaction, mean (SD)	90.9 (24.2)	92.7 (13.4)	83.6 (19.6)	0.079 ^∞^
Problem solution, mean (SD)	100 (0.0))	96.3 (8.0)	100 (0.0)	0.135 ^∞^
Perception of emotional change, mean (SD)	67.1 (19.4)	71.5 (12.6)	69.3 (10.2)	0.690 ^∞^

^∞^: Friedman’s test; SD: Standard deviation.

## Data Availability

The data presented in this study are available on request from the corresponding author.
